# Supplementary material for: The Systems Biology Research Tool: evolvable open-source software
Source: BMC Syst Biol. 2008 Jun 29;2:55. doi: 10.1186/1752-0509-2-55 (PMC2446383; doi:10.1186/1752-0509-2-55)
Supplement: Additional file 1 — SBRT Archive. An archive of the current version of the Systems Biology Research Tool. [file 1752-0509-2-55-S1.zip › sbrt-1.4.0/doc/developers_guide/api/sbrt/shell/text/math/DirEdgeFormatV1.html]

DirEdgeFormatV1


|  |  |  |
| --- | --- | --- |
| **Overview** \| **Package** \| **Class** \| **Use** \| **Tree** \| **Deprecated** \| **Index** \| **Help** | |  |
| **PREV CLASS**   **NEXT CLASS** | **FRAMES**    **NO FRAMES**     **All Classes** |  |
| SUMMARY: NESTED \| FIELD \| CONSTR \| METHOD | DETAIL: FIELD \| CONSTR \| METHOD |  |


---


## sbrt.shell.text.math Class DirEdgeFormatV1<N>

```
java.lang.Object
  sbrt.shell.text.math.DirEdgeFormatV1<N>
```

**Type Parameters:**: `N` - the node type.

**All Implemented Interfaces:**: Format, Formatter<DirectedGraph.Edge<N>>, DirEdgeFormat<N>, Parser<DirectedGraph.Edge<N>>, SimpleFormat<DirectedGraph.Edge<N>>

---

``` public final class DirEdgeFormatV1<N> extends java.lang.Object implements DirEdgeFormat<N> ```

This class is a concrete implementation of `DirEdgeFormat`.
The syntax used by this format is: `Source_Node -> Sink_Node`.
Whitespace surrounding the arrow is optional.

**Author:**
:   This class was written and documented by
    Jeremiah Wright while in the Wagner lab.

---

**Constructor Summary**

`DirEdgeFormatV1(SimpleFormat<N> nodeFormat)`
  
          Constructs a new directed edge format.


| **Method Summary** | |
| --- | --- |
| `java.lang.String` | `format(DirectedGraph.Edge<N> edge)`             Returns a formatted string representation of the provided edge. |
| `static DirEdgeFormatV1<java.lang.String>` | `getBasic()`             Returns a `DirEdgeFormatV1` that does not format or parse the nodes of the edges supplied to it. |
| `DirectedGraph.Edge<N>` | `parse(java.lang.String edge)`             Parses the provided string and returns its corresponding directed edge. |

**Methods inherited from class java.lang.Object**

`clone, equals, finalize, getClass, hashCode, notify, notifyAll, toString, wait, wait, wait`

**Methods inherited from interface sbrt.shell.text.Formatter**

`format`

**Constructor Detail**

### DirEdgeFormatV1

```
public DirEdgeFormatV1(SimpleFormat<N> nodeFormat)
```

:   Constructs a new directed edge format.

    **Parameters:**: `nodeFormat` - the format to use for nodes.


**Method Detail**

### getBasic

```
public static DirEdgeFormatV1<java.lang.String> getBasic()
```

:   Returns a `DirEdgeFormatV1` that does not
    format or parse the nodes of the edges supplied to it. In other
    words, it returns a `DirEdgeFormatV1` with
    the most basic possible functionality. Empty strings are not
    allowed however.

    :   **Returns:**: a `DirEdgeFormatV1` that does not format or parse the nodes of the edges supplied to it.

---


### format

```
public java.lang.String format(DirectedGraph.Edge<N> edge)
```

:   Returns a formatted string representation of the
    provided edge.

    :   **Parameters:**: `edge` - the edge to be formatted. **Returns:**: a formatted string representation of the provided edge.

---


### parse

```
public DirectedGraph.Edge<N> parse(java.lang.String edge)
```

:   Parses the provided string and returns its
    corresponding directed edge.

    :   **Specified by:**: `parse` in interface `Parser<DirectedGraph.Edge<N>>`
    :   **Parameters:**: `edge` - the string to be parsed. **Returns:**: the directed edge corresponding to the provided string.


---


|  |  |  |
| --- | --- | --- |
| **Overview** \| **Package** \| **Class** \| **Use** \| **Tree** \| **Deprecated** \| **Index** \| **Help** | |  |
| **PREV CLASS**   **NEXT CLASS** | **FRAMES**    **NO FRAMES**     **All Classes** |  |
| SUMMARY: NESTED \| FIELD \| CONSTR \| METHOD | DETAIL: FIELD \| CONSTR \| METHOD |  |


---
